# Factors influencing liberation from mechanical ventilation in coronavirus disease 2019: multicenter observational study in fifteen Italian ICUs

**DOI:** 10.1186/s40560-020-00499-4

**Published:** 2020-10-15

**Authors:** Lorenzo Gamberini, Tommaso Tonetti, Savino Spadaro, Gianluca Zani, Carlo Alberto Mazzoli, Chiara Capozzi, Emanuela Giampalma, Maria Letizia Bacchi Reggiani, Elisabetta Bertellini, Andrea Castelli, Irene Cavalli, Davide Colombo, Federico Crimaldi, Federica Damiani, Alberto Fogagnolo, Maurizio Fusari, Emiliano Gamberini, Giovanni Gordini, Cristiana Laici, Maria Concetta Lanza, Mirco Leo, Andrea Marudi, Giuseppe Nardi, Irene Ottaviani, Raffaella Papa, Antonella Potalivo, Emanuele Russo, Stefania Taddei, Carlo Alberto Volta, V. Marco Ranieri, the ICU-RER COVID-19 Collaboration, Marco Tartaglione, Valentina Chiarini, Virginia Buldini, Carlo Coniglio, Federico Moro, Nicola Cilloni, Lorenzo Giuntoli, Angela Bellocchio, Emanuele Matteo, Giacinto Pizzilli, Antonio Siniscalchi, Chiara Tartivita, Francesco Matteo, Annalisa Marchio, Igor Bacchilega, Laura Bernabé, Sonia Guarino, Elena Mosconi, Luca Bissoni, Lorenzo Viola, Tommaso Meconi, Vittorio Pavoni, Aline Pagni, Patrizia Pompa Cleta, Marco Cavagnino, Anna Malfatto, Angelina Adduci, Silvia Pareschi, Gabriele Melegari, Jessica Maccieri, Elisa Marinangeli, Fabrizio Racca, Marco Verri, Giulia Falò, Elisabetta Marangoni, Francesco Boni, Giulia Felloni, Federico Domenico Baccarini, Marina Terzitta, Stefano Maitan, Michele Imbriani, Paolo Orlandi, Giorgia Dalpiaz, Rita Golfieri, Federica Ciccarese, Antonio Poerio, Francesco Muratore, Fabio Ferrari, Martina Mughetti, Loredana Franchini, Ersenad Neziri, Marco Miceli, Maria Teresa Minguzzi, Lorenzo Mellini, Sara Piciucchi

**Affiliations:** 1 Department of Anaesthesia, Intensive Care and Prehospital Emergency, Ospedale Maggiore Carlo Alberto Pizzardi, Bologna, Italy; 2 Alma Mater Studiorum, Dipartimento di Scienze Mediche e Chirurgiche, Anesthesia and Intensive Care Medicine, Policlinico di Sant’Orsola, Università di Bologna, Bologna, Italy; 3 Department of Morphology, Surgery and Experimental Medicine, Section of Anaesthesia and Intensive Care University of Ferrara, Azienda Ospedaliero-Universitaria S. Anna, Via Aldo Moro, 8, 44121 Ferrara, Cona Italy; 4 Department of Anesthesia and Intensive Care, Santa Maria delle Croci Hospital, Ravenna, Italy; 5 Cardio-Anesthesiology Unit, Cardio-Thoracic-Vascular Department, S.Orsola Hospital, University of Bologna, Bologna, Italy; 6 Radiology Department, M.Bufalini Hospital, Cesena, Italy; 7 Alma Mater University, Department of Clinical, Integrated and Experimental Medicine (DIMES), Statistical Service, S. Orsola-Malpighi Hospital Bologna, Bologna, Italy; 8 Department of Anaesthesiology, University Hospital of Modena, Via del Pozzo 71, 41100 Modena, Italy; 9 Anaesthesia and Intensive Care Department, SS. Trinità Hospital, ASL, Novara, Italy; 10 Translational Medicine Department, Eastern Piedmont University, Novara, Italy; 11 Anaesthesia and Intensive Care Residency Program – Translational Medicine Department, Eastern Piedmont University, Novara, Italy; 12 Department of Anaesthesia, Intensive Care and Pain Therapy – Imola Hospital, Imola, Italy; 13 Anaesthesia and Intensive Care Unit, M. Bufalini Hospital, Cesena, Italy; 14 Division of Anesthesiology, Hospital S. Orsola Malpighi, Alma Mater Studiorum University of Bologna, Bologna, Italy; 15 Department of Anesthesia and Intensive Care, G.B. Morgagni-Pierantoni Hospital, Forlì, Italy; 16 Department of Anaesthesia and Intensive Care, Azienda Ospedaliera SS. Antonio e Biagio e Cesare Arrigo, Alessandria, Italy; 17 Department of Anaesthesia and Intensive Care, Infermi Hospital, Rimini, Italy; 18 Anaesthesia and Intensive Care Unit, Santa Maria Annunziata Hospital, Firenze, Italy; 19 Anaesthesia and Intensive Care Unit, Bentivoglio Hospital, Bentivoglio, Italy

**Keywords:** Coronavirus disease 2019, Intensive care, Mechanical ventilation, Outcomes, mortality, Respiration, artificial, ARDS

## Abstract

**Background:**

A large proportion of patients with coronavirus disease 2019 (COVID-19) develop severe respiratory failure requiring admission to the intensive care unit (ICU) and about 80% of them need mechanical ventilation (MV). These patients show great complexity due to multiple organ involvement and a dynamic evolution over time; moreover, few information is available about the risk factors that may contribute to increase the time course of mechanical ventilation.

The primary objective of this study is to investigate the risk factors associated with the inability to liberate COVID-19 patients from mechanical ventilation. Due to the complex evolution of the disease, we analyzed both pulmonary variables and occurrence of non-pulmonary complications during mechanical ventilation. The secondary objective of this study was the evaluation of risk factors for ICU mortality.

**Methods:**

This multicenter prospective observational study enrolled 391 patients from fifteen COVID-19 dedicated Italian ICUs which underwent invasive mechanical ventilation for COVID-19 pneumonia. Clinical and laboratory data, ventilator parameters, occurrence of organ dysfunction, and outcome were recorded. The primary outcome measure was 28 days ventilator-free days and the liberation from MV at 28 days was studied by performing a competing risks regression model on data, according to the method of Fine and Gray; the event death was considered as a competing risk.

**Results:**

Liberation from mechanical ventilation was achieved in 53.2% of the patients (208/391). Competing risks analysis, considering death as a competing event, demonstrated a decreased sub-hazard ratio for liberation from mechanical ventilation (MV) with increasing age and SOFA score at ICU admission, low values of PaO_2_/FiO_2_ ratio during the first 5 days of MV, respiratory system compliance (C_RS_) lower than 40 mL/cmH_2_O during the first 5 days of MV, need for renal replacement therapy (RRT), late-onset ventilator-associated pneumonia (VAP), and cardiovascular complications.

ICU mortality during the observation period was 36.1% (141/391). Similar results were obtained by the multivariate logistic regression analysis using mortality as a dependent variable.

**Conclusions:**

Age, SOFA score at ICU admission, C_RS_, PaO_2_/FiO_2_, renal and cardiovascular complications, and late-onset VAP were all independent risk factors for prolonged mechanical ventilation in patients with COVID-19.

**Trial registration:**

NCT04411459

## Introduction

A large proportion of patients with coronavirus disease 2019 (COVID-19) develop severe respiratory failure requiring admission to the intensive care unit (ICU) and about 80% of them need mechanical ventilation (MV) [1].

The reported mortality rate of mechanically ventilated patients with COVID-19 ranges from 30 to 97% [2–6]. Such a large variability may—at least in part—be due to the fact that a large portion of patients was still on mechanical ventilation in most of these studies. It is therefore relevant to identify the risk factors associated with a longer duration of mechanical ventilation. Of note, COVID-19 patients show great complexity due to multiple organ involvement, such as the lung, heart, kidney, and nervous system [7]. All these clinical features can contribute to the inability to liberate patients from MV. This may be particularly relevant since the majority of studies to date are focused mostly on admission clinical variables. Therefore, since COVID-19 is characterized by a dynamic evolution over time [8], few information is available on the risk factors that may contribute to increase the time course of mechanical ventilation, delaying the recovery to spontaneous ventilation.

The primary objective of this study is to investigate the risk factors associated with the inability to liberate COVID-19 patients from mechanical ventilation. Due to the complex evolution of the disease, we analyzed both pulmonary variables and the occurrence of non-pulmonary complications during mechanical ventilation.

## Methods

We conducted a prospective multicenter observational study in 15 ICUs. All consecutive patients with laboratory-confirmed SARS-CoV-2 infection admitted to participating dedicated COVID-19 ICUs from the 22nd of February through the 4th of May (the end of the nation-wide lockdown in Italy) 2020 were screened for eligibility. The clinical outcomes were monitored up to May 15, the final date of follow-up.

### Inclusion and exclusion criteria

Patients older than 18 years who received invasive mechanical ventilation were considered as eligible. Inclusion criteria were (a) SARS-CoV-2 infection confirmed by real-time reverse transcription-polymerase chain reaction assays from either nasal swabs or lower respiratory tract samples [9] and (b) use of invasive mechanical ventilation at any time of the clinical course. Exclusion criteria were (a) not a laboratory-confirmed SARS-CoV-2, (b) use of extracorporeal membrane oxygenation (ECMO) and/or extracorporeal CO_2_ removal (ECCO_2_R), (c) use of non-invasive ventilation during the entire clinical course, and (d) cardiac arrest before intubation.

Patients with missing data for the variables of interest (comorbidities, length of mechanical ventilation, ventilatory parameters during the first 5 days, ICU complications, and discharge information) were not included in the final analysis.

The study was approved by the Institutional Review Board (IRB) of the study coordinator center (Maggiore Hospital, Bologna, Italy, approval number: 273/2020/OSS/AUSLBO) and by each institutional review committee of the participating hospitals. Informed consent was partially waived according to the approval of the local ethics committee, and researchers analyzed anonymized individual data. The study was registered in ClinicalTrials.gov (NCT04411459).

### Data collection

A list of clinical variables was defined before the initiation of the study using a priori designed case report forms. Data were collected by one investigator of each hospital in an electronic case report form developed by YGHEA, CRO division of Ecol Studio SPA (Bologna Operational Headquarters), and hosted by ACTide Nubilaria (Novara, Italy). Collected data comprised demographic data, information on clinical symptoms, or signs at presentation, underlying comorbidities, laboratory findings, imaging studies, and respiratory parameters before the intubation and ventilator setting during the first 5 days of mechanical ventilation (e.g., positive end-expiratory pressure (PEEP), plateau pressure (Pplat), static compliance (C_RS_), PaO_2_/FiO_2_ ratio), and clinical outcomes. Additional details on collected variables are available in the online supplement.

### Outcome variables

Ventilator-free days at day 28 was the primary outcome variable. Risk factors associated with ICU mortality were also assessed.

### Definitions

Mechanical ventilation was considered invasive if delivered through an endotracheal tube or a tracheostomy. The duration of mechanical ventilation was defined as the time elapsed from intubation to extubation or successful disconnection from mechanical ventilation for tracheostomized patients. Extubation failure was defined as the need for reintubation within 48 h, and the time from failed extubation to reintubation was recorded.

ARDS was classified into three increasing levels of oxygenation failure into mild, moderate, and severe, according to the Berlin definition of ARDS [10].

For respiratory system compliance, we chose a cut-off of 40 ml/cmH_2_O for discriminating between higher and lower compliance. This cut-off was previously proposed in the Berlin definition of ARDS as an ancillary variable for defining the most severe cases [10].

Ventilator-free days (VFDs) were defined as a time frame of 28 days from intubation. For intubated patients, in case of reintubation within 28 days, VFDs were counted from the last successful extubation. The use of non-invasive ventilation (NIV) after extubation was not considered as a ventilation period. Finally, zero VFDs were assigned to 28-day non-survivors, regardless of their intubation status [11]. In tracheostomized patients, interval disconnections were not counted and VFDs started after the last successful disconnection from mechanical ventilation [11].

### Statistical analysis

Data were analyzed using Stata/SE 15.1 (College Station, Texas, USA); continuous variables were expressed as the median and interquartile range (IQR); comparisons between continuous variables were performed with Mann-Whitney *U* test; categorical variables were expressed as numbers and percentages and compared using chi-squared test.

The liberation from MV at 28 days was studied by performing a competing risks regression model on data, according to the method of Fine and Gray; the event death was considered as a competing risk [11].

Model building was performed by means of a variable selection based on an initial screening using univariate analysis with *p* < 0.2 criteria, then a stepwise selection with entry criteria = 0.05 and stay criteria = 0.1.

Estimates of coefficients in the model are reported as sub-hazard ratios along with the graphs of the cumulative incidence function of liberation from MV on the basis of either static compliance of the respiratory system or PaO_2_/FiO_2_ ratio range adjusted for the other covariates introduced into the multivariate model.

Univariate and multivariable logistic regression analyses were performed in order to evaluate factors associated with death during ICU stay. Screened risk variables and model building were the same as for competing risks regression. The area under the receiver operator characteristic (ROC) was reported.

In all analyses, the standard errors were adjusted considering enrolling centers as clusters; therefore, assuming that observations were independent across different hospitals but not necessarily within the same center. All *p* values refer to two-tailed tests of significance and *p* < 0.05 was considered significant.

## Results

### Population

Over the study period, 607 patients were screened for eligibility. Patients excluded for admission for other causes than respiratory failure, use of ECMO/ECCO2R, use of non-invasive ventilation during the entire clinical course, and cardiac arrest before intubation were 48, 11, 67, and 6, respectively. Eighty-four patients had missing data for the variables of interest and were not included for the final analysis. Three hundred ninety-one patients were therefore included in the final analysis (figure S1, online supplement).

Main demographics, comorbidities, and clinical characteristics at ICU admission are detailed in Table 1. The patients were predominantly male (300/391, 77%), with a median [IQR] age of 66 years [59-72]. Hypertension was the most common comorbidity (222/391, 57%).

**Table 1:** Main patients characteristics

	Total population (***n*** = 391)	Successful liberation from MV 28 days (***n*** = 165)	Unsuccessful liberation from MV 28 days (***n*** = 226)	***p***
**Variables**				
Age - year (IQR)	66 (59–72)	64 (56–70)	68 (62–74)	**< 0.001**
Sex - male – no (%)	300 (76.7%)	121 (73.3%)	179 (79.2%)	0.175
BMI - median (IQR)	28 (26–31)	28 (26–31)	28 (26–31)	0.918
SAPS II score	38 (31–46)	35 (28–42)	39 (33–48)	**< 0.001**
SOFA score at ICU admission	5 (3–7)	4 (3–6)	6 (4–8)	**< 0.001**
**Comorbidities**				
Hypertension - no (%)	222 (56.8%)	79 (47.9%)	143 (63.3%)	**0.003**
Chronic ischemic heart disease - no (%)	35 (9.0%)	12 (7.3%)	23 (10.2%)	0.416
Chronic kidney disease (CKD)	26 (6.6%)	8 (4.8%)	18 (8.0%)	0.362
CKD - patients in dialisys - no (%)	4 (1.0%)	1 (0.6%)	3 (1.3%)	
COPD - no (%)	27 (6.9%)	11 (6.7%)	16 (7.1%)	0.964^*a*^
COPD - home oxygen therapy/CPAP - no (%)	2 (0.5%)	1 (0.6%)	1 (0.4%)	
Diabetes - no (%)	85 (21.7%)	28 (17.0%)	57 (25.2%)	0.067
Chronic liver disease (MELD > 10) - no (%)	3 (0.8%)	1 (0.6%)	2 (0.9%)	1.000
Active cancer - no (%)	7 (1.8%)	0 (0%)	7 (3.1%)	0.058
Immunosuppressive therapy - no (%)	10 (2.6%)	1 (0.6%)	9 (4.0%)	0.078
Smoker status (*n* = 213) ^*b*^				
Current - no (%)	13 (6.1%)	8 (8.1%)	5 (4.4%)	0.507^*a*^
Previous - no (%)	66 (31.0%)	29 (29.3%)	37 (32.5%)	
**Characteristics before ICU admission**				
Time from symptoms onset to hospital admission (*n* = 327)^b^ - d (IQR)	7 (4–9)	7 (4–9)	7 (4–10)	0.480
Time from hospital admission to ICU admission - d (IQR)	2 (1–5)	2 (1–5)	2 (0–5)	0.436
Time from hospital admission to intubation - d (IQR)	2 (1–5)	2 (1–5)	2 (1–6)	0.389
Ward of admission				
Emergency department - no (%)	70 (17.9%)	20 (12.1%)	50 (22.1%)	**0.04**^*a*^
Medical ward - no (%)	184 (47.1%)	82 (49.7%)	102 (45.1%)	
Other ICU - no (%)	137 (35.0%)	63 (38.2%)	74 (32.7%)	
High flow nasal oxygen therapy before intubation (*n* = 362)^b^ - no (%)	31 (8.5%)	14 (9.1%)	17 (8.1%)	0.880
CPAP/Non-invasive ventilation trial before intubation (*n* = 363)^b^ - no (%)	254 (70%)	117 (70.9%)	137 (60.6%)	**0.029**
Duration of CPAP/NIV trial (*n* = 363)^b^				
< 12 h - no (%)	56 (22.1%)	24 (20.5%)	32 (23.5%)	0.051^*a*^
12–24 h - no (%)	63 (24.9%)	35 (29.9%)	28 (20.6%)	
24–48 h - no (%)	44 (17.4%)	25 (21.4%)	19 (14.0%)	
> 48 h - no (%)	90 (35.6%)	33 (28.2%)	57 (41.9%)	
PaO_2_/FiO_2_ ratio before intubation (*n* = 297)^b^	94 (75–119)	96 (71–125)	91 (77–116)	0.544

Abbreviations: *IQR* interquartile range, *BMI* body mass index, *SAPS* simplified acute physiology score, *SOFA* Sequential Organ Failure Assessment Score, *CKD* chronic kidney disease, *COPD* chronic obstructive pulmonary disease, *CPAP* continuous positive airway pressure, *MELD* model for end-stage liver disease, *NIV* non-invasive ventilation, *PaO*_*2*_ arterial oxygen partial pressure, *FiO*_*2*_ inspired fraction of oxygen

^a^*p* value referred to Pearson’s chi-square test performed on the contingency table represented

^b^Incomplete data due to transfer from other ICUs without complete medical records

Non-invasive ventilation or CPAP was applied in 254 patients (65%) before tracheal intubation. Fifty-four of them (21% of patients receiving NIV/CPAP) received NIV in the ICU for more than 24 h. Patients were intubated after a median of 2 [1–5] days from hospital admission.

Before starting MV, the median PaO_2_/FiO_2_ ratio was 94 [75 - 119] mmHg. Initial respiratory parameters showed a median positive end-expiratory pressure (PEEP) of 12 [10 - 14] cmH_2_O which resulted in a static respiratory system compliance (C_RS_) of 38 mL/cmH_2_O [31 - 47]. During the first 5 days of mechanical ventilation, the lowest recorded PaO_2_/FiO_2_ ratio was 100 [76 – 132]. Almost all patients (96.2%) had a PaO_2_/FiO_2_ ratio lower than 200. Tracheostomy was performed in 224 patients (57.3%) with a median time from orotracheal intubation to tracheostomy of 9 [5 - 12] days. Two-hundred fifty-eight patients (66.0%) were treated with at least one cycle of prone positioning and 365 patients (93%) received a continuous infusion of neuromuscular blocking agents (NMBA) for at least 24 h (see Table S1, online supplement for further details about adjunctive treatments) (Table 2).

**Table 2:** Main results—ICU ventilation data, complications, and outcomes

	All patients	Successful liberation from MV 28d	Unsuccessful liberation from MV 28d	
**Initial ventilatory variables** ^*a*^	***n =*** **240** ^*a*^	***n*** **= 93** ^*a*^	***n*** **= 147** ^*a*^	***p***
Tidal volume set - mL/kg IBW (IQR)	7.1 (6.5–7.8)	7 (6.5–7.9)	7.1 (6.4–7.7)	0.932
PEEP set - cmH_2_O (IQR)	12 (10–14)	12 (10–14)	12 (10–14)	0.466
Pplat - cmH_2_O (IQR)	25 (22–27)	25 (22–27)	25 (22–28)	0.662
C_RS_ in supine position - mL/cmH_2_O (IQR)	38 (32–47)	39 (32–48)	37 (31–47)	0.297
C_RS_ in supine position < 40 mL/cmH_2_O – no (%)	132 (55%)	49 (52.7%)	83 (56.5%)	0.567
**Mechanical ventilation - first 5 days**	***n*** **= 391**	***n*** **= 165**	***n*** **= 226**	***p***
Highest FiO_2_ set for at least 12 h - % (IQR)	70 (60–80)	70 (60–80)	75 (60–90)	**< 0.001**
Lowest PaO_2_/FiO_2_ ratio in supine position - (IQR)	100 (76–132)	113 (90–145)	94 (68–123)	**< 0.001**
PaO_2_/FiO_2_ class				**< 0.001**^*b*^
200–300 - no (%)	15 (3.8%)	6 (3.6%)	9 (4.0%)	
100–200 - no (%)	195 (49.9%)	106 (64.2%)	89 (39.4%)	
< 100 - no (%)	181 (46.3%)	53 (32.1%)	128 (56.6%)	
Lowest static C_RS_ - mL/cmH_2_O (IQR)	37 (30–45)	40 (33–47)	35 (30–43)	**< 0.001**
C_RS_ < 40 mL/cmH_2_O - no (%)	225 (57.5%)	77 (46.7%)	148 (65.5%)	**< 0.001**
**ICU stay variables**	***n*** **= 391**	***n*** **= 165**	***n*** **= 226**	***p***
Liberation from mechanical ventilation - no (%)	208 (53.2%)	165 (100%)	43 (19%)	–
VFD 28 days - d (IQR)	14 (9–19)	14 (9–19)	0	–
Duration of MV - d (IQR)	16 (10–27)	14 (9 –19)	20 (11–33)	**< 0.001**
Alive^c^ (*n* = 250) – d (IQR)	17 (10–30)	14 (9–19)	35 (29–46.5)	**< 0.001**
Extubation - no (%)	89 (22.8%)	81 (49%)	8 (3.5%)	**< 0.001**
Tracheotomy - no (%)	224 (57.3%)	83 (50.3%)	141 (62.4%)	**0.022**
Time from first tracheal intubation to tracheotomy - d (IQR)	9 (5–12)	7 (4–11)	9 (6–13)	**0.002**
Length of ICU stay - days (IQR)	20 (13–32)	18 (14–27)	21 (11–38)	0.111
Alive^d^ (*n* = 250) - days (IQR)	24 (15–38)	18 (14–27)	42 (32–51)	**< 0.001**
Dead (*n* = 141) - days (IQR)	15 (8–22)	0	15 (8–22)	–
**Post extubation events**^c^	***n*** **= 89**	***n*** **= 81**	***n*** **= 8**	***p***
Extubation failure within 48 h - no (%)	9 (10.1%)	5 (6.1%)	4 (50%)	**< 0.001**
Extubation failure over 48 h - no (%)	7 (7.9%)	3 (3.7%)	4 (50%)	**< 0.001**
NIV/CPAP after extubation - no (%)	51 (57.3%)	43 (53%)	8 (100%)	**0.017**
HFNO after extubation - no (%)	5 (5.6%)	9 (11%)	1 (12.5%)	0.980
**ICU Complications**	***n*** **= 391**	***n*** **= 165**	***n*** **= 226**	***p***
Cardiovascular - no (%)	66 (16.9%)	10 (6.1%)	56 (24.8%)	**< 0.001**
Neurologic - no (%)	25 (6.4%)	7 (4.2%)	18 (8.0%)	0.137
Gastroenteric - no (%)	20 (5.1%)	6 (3.6%)	14 (6.2%)	0.257
Need for renal replacement therapy - no (%)	76 (19.4%)	13 (7.9%)	63 (27.9%)	**< 0.001**
Early onset VAP - no (%)	76 (19.4%)	29 (17.6%)	47 (20.8%)	0.427
Lateonset VAP - no (%)	175 (44.8%)	49 (29.7%)	126 (55.8%)	**< 0.001**
Non-pulmonary infections - no (%)	112 (28.6%)	35 (21.2%)	77 (34.1%)	**0.005**
Still in ICU at the end of observation - no (%)	39 (9.7%)	4 (2.4%)	35 (15.5%)	**< 0.001**
ICU mortality^d^ - no (%)	141 (36.1%)	0 (0%)	141 (62.4%)	**< 0.001**

Abbreviations: *IBW* ideal body weight, *PEEP* positive end-expiratory pressure, *Pplat* plateau pressure, *C*_*RS*_ respiratory system compliance measured in a supine position, *FiO*_*2*_ inspired fraction of oxygen, *PaO*_*2*_ arterial oxygen partial pressure, *MV* mechanical ventilation, *NIV* non-invasive ventilation, *CPAP* continuous positive airway pressure, *HFNO* high flow nasal oxygen, *VFD* ventilator-free days, *VAP* ventilator-associated pneumonia

^a^Incomplete data due to transfer from other ICUs without complete medical records

^b^*p* value referred to Pearson’s chi-square test performed on the contingency table represented ^c^The percentages of extubation failures and NIV/CPAP/HFNO use after extubation are referred to the total number of the patients extubated

^d^Patients discharged alive from ICU or still in ICU at the end of observation

In this cohort, 216 patients (55.2%) experienced at least one non-pulmonary complication, the most frequent being acute kidney injury needing renal replacement therapy (76 patients,19.4%) and cardiovascular complications (66 patients, 16.9%).

Among pulmonary complications, the most frequent was the late-onset ventilator-associated pneumonia (175 patients, 44.8%). Table S2 (online supplement) describes the specific complications observed.

During the period of observation, liberation from MV was achieved in 208 patients (53.2%) after a mean duration of MV of 14 [9 – 19] days. Clinical characteristics, as well as respiratory parameters and occurrences of non-pulmonary organ failure, are reported in Table 2. Initial ventilatory variables did not show differences between groups. During the first 5 days of MV, both PaO_2_/FiO_2_ ratio and C_RS_ were significantly higher in patients who achieved a successful liberation.

Competing risks analysis demonstrated a decreased sub-hazard ratio (SHR) for liberation from mechanical ventilation with increasing age and SOFA score at ICU admission, with decreasing lowest PaO_2_/FiO_2_ ratio and C_RS_ lower than 40 mL/cmH_2_O during the first 5 days of MV, need for renal replacement therapy (RRT) during ICU stay, late-onset ventilator-associated pneumonia (VAP), and cardiovascular complications. Univariate and multivariate analyses for liberation from mechanical ventilation are presented in Table 3.

**Table 3:** Fine and Gray’s competing-risks analysis

	Univariate analysis			Multivariate analysis		
Variable	SHR	95% CI	***p***	SHR	95% CI	***p***
Age	0.968	0.959–0.978	< 0.001	0.979	0.966–0.992	**0.002**
Sex (M)	0.716	0.491–1.044	0.083	–		
BMI	0.999	0.974–1.024	0.922	–		
SOFA score at ICU admission	0.836	0.788–0.887	< 0.001	0.867	0.792–0.949	**0.002**
SAPS II score	0.973	0.959–0.987	< 0.001	–		
Hypertension	0.648	0.498–0.843	0.001	–		
Chronic ischemic heart disease	0.760	0.473–1.221	0.257	–		
COPD (oxygen therapy/CPAP)	0.982	0.219–4.407	0.981	–		
Chronic kidney disease (CKD)	0.692	0.303–1.582	0.383	–		
CKD –dialisys	0.532	0.061–4.640	0.568	–		
Diabetes	0.703	0.459–1.078	0.106	–		
Chronic liver disease (MELD > 10)	0.853	0.224–3.239	0.815	–		
Renal replacement therapy during ICU stay	0.274	0.142–0.531	< 0.001	0.381	0.220–0.660	**0.001**
Early onset VAP	0.844	0.600–1.188	0.331	–		
Late-onset VAP	0.357	0.251–0.510	< 0.001	0.283	0.197–0.407	**< 0.001**
Lowest PaO_2_/FiO_2_ within 5 days^a^	1.729	1.312–2.280	< 0.001	1.631	1.316–2.023	**< 0.001**
C_RS_ < 40 mL/cmH_2_O within 5 days	0.538	0.350–0.826	0.005	0.488	0.317–0.752	**0.001**
Cardiovascular complications	0.253	0.175–0.367	< 0.001	0.277	0.181–0.423	**< 0.001**
Neurologic complications	0.550	0.236–1.283	0.167	0.469	0.207–1.061	0.069
Gastrointestinal complications	0.578	0.374–0.894	0.014	–		
Extrapulmonary infections	0.533	0.326–0.872	0.012	–		

Notes: Event of interest—liberation from mechanical ventilation. Competing event- death. Observation time: 28 days

Abbreviations: *SHR* subhazard ratio, *BMI* body mass index, *SAPS* simplified acute physiology score, *SOFA* Sequential Organ Failure Assessment Score, *COPD* chronic obstructive pulmonary disease, *CKD* chronic kidney disease, *MELD* model for end-stage liver disease, *PaO*_*2*_ arterial oxygen partial pressure, *FiO*_*2*_ inspired fraction of oxygen, *C*_*RS*_ respiratory system compliance

^a^SHR calculated per 100 points increase of PaO_2_/FiO_2_ ratio

**Fig. 1:**
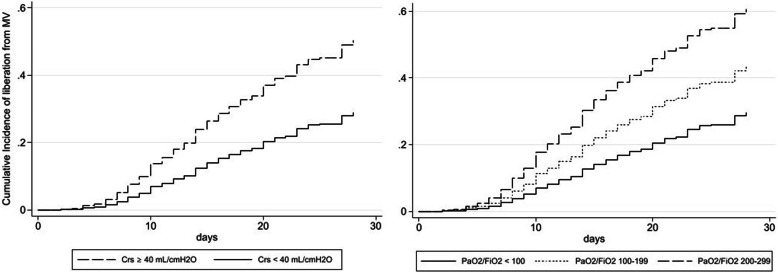
Cumulative incidence function of liberation from mechanical ventilation on the divided for either static compliance of the respiratory system (C_RS_) or PaO_2_/FiO_2_ ratio range and adjusted for the other covariates introduced into the multivariate model of the competing-risks regression

Figure 1 shows the cumulative incidence function of 28-day liberation from mechanical ventilation divided for classes of worst C_RS_ or PaO_2_/FiO_2_ ratio observed within the first 5 days of MV and adjusted for the significant covariates defined in Table 3.

### Secondary outcome

Multivariate logistic regression analysis using mortality as a dependent variable showed that increasing age, higher SOFA score at ICU admission, need for renal replacement therapy during ICU stay, cardiovascular complications, lower PaO_2_/FiO_2_ ratio, and low C_RS_ during the first 5 days of invasive MV were independently associated to ICU mortality. Table 4 shows the results of univariate and multivariate analyses on the abovementioned variables (see Figure S2 online supplement for the ROC curve).

**Table 4:** Multivariate logistic regression analysis, dependant variable death during ICU stay

	Univariate logistic regression analysis			Multivariate logistic regression analysis		
Variable	HR	95% C.I.	***p***	HR	95% C.I.	***p***
Age	1.067	1.029–1.107	< 0.001	1.073	1.032–1.116	**< 0.001**
Sex (M)	1.120	0.735–1.709	0.597	–		
BMI	0.992	0.945–1.042	0.761	–		
SOFA score at ICU admission	1.215	1.095–1.349	< 0.001	1.142	1.007–1.295	**0.038**
SAPS II score	1.042	1.022–1.063	< 0.001	–		
Hypertension	1.906	1.182–3.073	0.008	–		
Chronic ischemic heart disease	1.053	0.556–1.992	0.875	–		
COPD (oxygen therapy/CPAP)	1.454	0.060–35.34	0.818	–		
Chronic kidney disease (CKD)	1.599	0.706–3.617	0.260	–		
CKD – dialisys	5.595	0.512–61.132	0.158	–		
Diabetes	3.583	0.793–16.195	0.097	–		
Chronic liver disease (MELD > 10)	3.583	0.793–16.195	0.097	–		
Renal replacement therapy during ICU stay	2.885	2.265–3.675	< 0.001	2.115	1.037–4.310	**0.039**
Early onset VAP	1.574	0.797–3.108	0.192	–		
Late-onset VAP	1.190	0.787–1.812	0.410	–		
Lowest PaO_2_/FiO_2_ within 5 days^a^	0.396	0.248–0.631	< 0.001	0.383	0.239–0.613	**< 0.001**
C_RS_ < 40 mL/cmH_2_O within 5 days	2.844	2.221–3.642	< 0.001	3.193	2.525–4.040	**< 0.001**
Cardiovascular complications	4.701	2.807–7.874	< 0.001	4.600	2.474–8.554	**< 0.001**
Neurologic complications	0.673	0.233–1.947	0.465	–		
Gastrointestinal complications	0.749	0.288–1.952	0.555	–		
Extrapulmonary infections	0.877	0.488–1.578	0.662	–		

Notes: AUC of the ROC curve for the multivariate model: 0.818

Abbreviations: *HR* hazard ratio, *C.I.* confidence interval, *BMI* body mass index, *SAPS* simplified acute physiology score, *SOFA* Sequential Organ Failure Assessment Score, *COPD* chronic obstructive pulmonary disease, *CKD* chronic kidney disease, *MELD* model for end-stage liver disease, *VAP* ventilator-associated pneumonia, *PaO*_*2*_ arterial oxygen partial pressure, *FiO*_*2*_ inspired fraction of oxygen, *C*_*RS*_ respiratory system compliance

^a^Per 100 points increase of PaO_2_/FiO_2_ ratio

## Discussion

The main findings of this prospective analysis are (a) invasively ventilated COVID-19 patients exhibited both a long duration of mechanical ventilation and ICU length of stay; (b) age, SOFA score, PaO_2_/FiO_2_ ratio, C_RS_, acute kidney injury (requiring renal replacement therapy), late pulmonary infections, and cardiovascular complications were all independent risk factors for prolonged mechanical ventilation; and (c) our patients’ population had PaO_2_/FiO_2_ and C_RS_ values resembling those of “classical” (i.e., non-COVID-19 related) ARDS.

Most observational studies published until now on COVID-19 patients investigated the risk factors for hospital mortality, whereas our aim was to focus on risk factors responsible for prolonged mechanical ventilation.

The risk factors we identified may be classified into three main categories: demographics and baseline severity, physiology, and complications during the ICU stay.

Among demographical variables, increasing age was significantly associated with a higher duration of MV and ICU mortality and this is in line with recent literature on COVID-19 patients [12], but also on “classical” ARDS patients [2, 13]. As for baseline severity, SOFA score at ICU admission, but not SAPS II score, was inversely associated to an increased SHR for liberation from mechanical ventilation at 28 days and directly associated to death in the multivariate competing risk and logistic analyses [2, 14, 15].

Considering the physiological characteristics of the patients, most of them exhibited a reduced static compliance. A C_RS_ < 40 mL/cmH_2_O was previously proposed in the Berlin definition of ARDS as an ancillary variable for defining the most severe cases [10]; moreover, this cut-off has been recently proposed to identify a more severe phenotype of COVID-19 [16]. In our patients, C_RS_ < 40 mL/cmH_2_O was independently associated with both prolonged mechanical ventilation (Fig. 1) and mortality. In this regard, we also showed that 55% of patients had C_RS_ < 40 mL/cmH_2_O at starting of MV, while 57% of patients experienced C_RS_ < 40 mL/cmH_2_O during the first 5 days. Of note, C_RS_ after intubation did not differ between patients who were or not liberated from MV but the percentage of patients with C_RS_ < 40 mL/cmH_2_O within 5 days was significantly different. Although our data do not allow definite conclusions, we can underline that a C_RS_ < 40 mL/cmH_2_O possibly represents a marker of worse ventilatory outcome in COVID-19 patients.

As expected from current knowledge on COVID-19 [17], oxygenation was compromised and a high FiO_2_ (median value 70%) was needed. Further, the lowest PaO_2_/FiO_2_ ratio observed during the first 5 days of MV was a limiting factor for liberation from mechanical ventilation, as it has been demonstrated in “classical” ARDS patients [2, 18, 19].

The debate on whether COVID-19 patients have a special form of ARDS is still ongoing [17, 20], but recent papers focused on COVID-19-ARDS pathophysiology report values of oxygenation (P/F ratio) and compliance similar to those of “classical” ARDS [12, 19, 21–23]. The totality of our patients matches oxygenation criteria for ARDS, most of them exhibit impaired lung mechanics (low static compliance) and need treatment with relatively high levels of PEEP (median 12 cmH_2_O) [24] and FiO_2_ (median 70%) to warrant adequate oxygenation, all characteristics common to “classical” ARDS [25]. This may explain why most of the risk factors for prolonged mechanical ventilation we have identified are common to those of ARDS due to other causes. Of course, some features of ARDS due to COVID-19 seem to be peculiar and particularly important in determining the outcome. One of the main histological findings in COVID-19 ARDS is a significant endothelial involvement due to viral antigen exposure and cytokine activation that leads to inflammation activation and possible cytokine storm, mostly through the activation of an angiotensin-converting enzyme 2 (ACE2) receptor (expressed in pulmonary endothelial cells, alveolar epithelial type II cells, heart, intestine, and kidney). This histological damage can in part explain the prothrombotic state of COVID-19 patients, and it has been observed that higher D-dimer levels correlate with the severity of the disease and the in-hospital mortality [26]; moreover, very high mortality rates have been observed if high D-dimer levels are combined with low static compliance values [22].

The third category of risk factors for prolonged mechanical ventilation we identified is represented by the complications during ICU stay. Indeed, the survival outcome of mechanically ventilated patients depends not only on baseline characteristics but also on further development of complications. These can be further subdivided into non-pulmonary and pulmonary. Among non-pulmonary complications, the most frequent were acute kidney injury needing renal replacement therapy (19%) and cardiovascular events (17%).

Acute kidney injury has already been described in COVID-19 patients; although pathophysiological mechanisms should be further characterized, SARS-CoV-2 seems to affect the kidney directly or indirectly [27, 28]. The cause of kidney involvement in COVID-19 is likely to be multifactorial: endothelial damage due to virus particles, an ACE2-dependent pathway causing cellular dysfunction, and immune response dysregulation in a hypercoagulability and endotheliitis state are probably the most important contributor to acute kidney injury [29–31]. Patients developing acute kidney injury during ICU stay frequently have comorbidities [32, 33], and they usually have less favorable outcome [33] and easily develop complications related to fluid overload [34]; consequently, acute kidney injury is a known risk factor for prolonged mechanical ventilation in critically ill patients, regardless of the underlying disease [35, 36].

A high rate of cardiovascular events in COVID-19 patients has already been reported [37–39]; in particular, myocardial injury was described in 7.2% of patients overall and in 22% of patients requiring ICU admission [40], a percentage similar to the one recorded in our study (17%). The pathophysiologic mechanisms are still unclear, the most probably being (a) the direct viral action on the myocardium (i.e., SARS-CoV-2 myocarditis) and (b) the cardiomyopathy caused by cytokine storm (similar to septic cardiomyopathy) [37]. Our data show that cardiovascular complications are a strong predictor not only of mortality, but also of delayed separation from mechanical ventilation.

Among pulmonary complications, infections played a major role. Late-onset ventilator-associated pneumonia (VAP) had a relevant incidence (44.8%) in our patients, considerably higher than that observed in other studies on “classical” ARDS [41]. This result could be explained by the frequent use of immunomodulatory agents as adjunctive therapies [42] or to SARS-CoV-2 infection per se [43] (see supplement Table 1). Although the description of incidence, risk factors, and microbiology of late-onset VAP goes beyond the aims of this study, we believe that this aspect can be of clinical relevance, deserving future studies.

Besides the high incidence of VAP, the high rate of tracheostomy may be related to the length of MV and the relatively high incidence of reintubation. The median duration of invasive ventilation in our COVID-19 patients was 16 days, and this is in line with other recent reports, showing a range of 10–18 days [12, 19, 21, 23]; meanwhile, in “classical” ARDS durations between 6 days for mild and 11 days for severe ARDS have been reported [2]. The reason for this discrepancy has to be further investigated, but we can formulate some hypotheses: first, ARDS patients in the LUNG-SAFE cohort were much more heterogeneous than our cohort of viral pneumonias due to SARS-CoV-2 infection; second, in COVID-19 patient, the involvement of the central nervous system [44], heart [45], and kidney [30] is frequent, thus explaining the longer need for mechanical ventilation and the noticeably long ICU length of stay (median 20 days overall, 24 days in surviving patients) we recorded in our population. Indeed, 57.3% of the patients underwent tracheostomy within a median of 9 days [5–12] from intubation. Although a precise definition of “early” and “late” tracheostomy is still lacking, our patients can be placed in the upper range of early tracheostomy, according to previous trials [46, 47]. To date, no guidelines exist on the optimal timing of tracheostomy in COVID-19 patients, although an expert consensus suggests delaying tracheostomy at least 10 days after intubation [48].

This study has some limitations. First, although data collection was prospective, ventilatory treatment and weaning were not standardized among participating centers, thus adding potential confounding factors. Second, for many variables, we asked the participating centers to collect the lowest values in the first 5 days of ICU stay, thus possibly missing valuable data on the precise time-course of these variables. Third, various experimental COVID-19 therapies were tested in different centers during the conduction of this study (see Table S1, online supplement). In order to control for the center-related effects, clusterization was adopted for statistical analysis. Finally, 22 patients had not completed the observation period for outcome measures, either because they were still in the ICU at the end of the observation period or because they were transferred for logistical reasons to other non-participating ICUs; in any case, censoring before the end of observation was taken into account by competing risks regression analysis.

Even though regional and national healthcare systems were experiencing high levels of stress at the time of data collection, no rationing of healthcare resources [49] was in place in any participating center. Accordingly, although organizational issues do not fall within the scope of this paper, we can exclude that organizational issues may have contributed to unfavorable outcomes.

Despite these limitations, to the best of our knowledge, this is the first study to elucidate the risk factors associated with prolonged mechanical ventilation in COVID-19 patients. Our findings may help clinicians predict the risk for prolonged mechanical ventilation. Once patients with multiple risk factors are identified, clinicians should consider the possibility of a high failure rate of life-sustaining interventions and discuss the possible shift towards a palliative approach. Such a decision should be rigorous and comprehensive of all clinical information. However, additional large-scale studies are still warranted to validate our findings.

## Conclusions

Patients with COVID-19 exhibited a high risk of failure from MV liberation at 28 days. Age, SOFA score, PaO_2_/FiO_2_ ratio, C_RS_, need for renal replacement therapy, late-onset VAP, and cardiovascular complications were all independent risk factors for prolonged mechanical ventilation.

## Supplementary information

**Additional file 1:.** .

## Data Availability

The datasets used and/or analyzed during the current study are available from the corresponding author on reasonable request.

## Abbreviations

MV: Mechanical ventilation
ICU: Intensive care unit
C_RS_: Static respiratory system compliance
ECMO: Extracorporeal membrane oxygenation
ECCO_2_R: Extracorporeal CO_2_ removal
RRT: Renal replacement therapy
SOFA score: Sequential Organ Failure Assessment
SAPS II score: Simplified Acute Physiology Score
VAP: Ventilator-associated pneumonia
