# Correction to: Factors influencing liberation from mechanical ventilation in coronavirus disease 2019: multicenter observational study in fifteen Italian ICUs

**DOI:** 10.1186/s40560-020-00514-8

**Published:** 2020-12-17

**Authors:** Lorenzo Gamberini, Tommaso Tonetti, Savino Spadaro, Gianluca Zani, Carlo Alberto Mazzoli, Chiara Capozzi, Emanuela Giampalma, Maria Letizia Bacchi Reggiani, Elisabetta Bertellini, Andrea Castelli, Irene Cavalli, Davide Colombo, Federico Crimaldi, Federica Damiani, Alberto Fogagnolo, Maurizio Fusari, Emiliano Gamberini, Giovanni Gordini, Cristiana Laici, Maria Concetta Lanza, Mirco Leo, Andrea Marudi, Giuseppe Nardi, Irene Ottaviani, Raffaella Papa, Antonella Potalivo, Emanuele Russo, Stefania Taddei, Carlo Alberto Volta, V. Marco Ranieri, the ICU-RER COVID-19 Collaboration

**Affiliations:** 1 Department of Anaesthesia, Intensive Care and Prehospital Emergency, Ospedale Maggiore Carlo Alberto Pizzardi, Bologna, Italy; 2 Alma Mater Studiorum, Dipartimento di Scienze Mediche e Chirurgiche, Anesthesia and Intensive Care Medicine, Policlinico di Sant’Orsola, Università di Bologna, Bologna, Italy; 3 Department of Morphology, Surgery and Experimental Medicine, Section of Anaesthesia and Intensive Care University of Ferrara, Azienda Ospedaliero-Universitaria S. Anna, Via Aldo Moro, 8, 44121 Ferrara, Cona Italy; 4 Department of Anesthesia and Intensive Care, Santa Maria delle Croci Hospital, Ravenna, Italy; 5 Cardio-Anesthesiology Unit, Cardio-Thoracic-Vascular Department, S.Orsola Hospital, University of Bologna, Bologna, Italy; 6 Radiology Department, M.Bufalini Hospital, Cesena, Italy; 7 Alma Mater University, Department of Clinical, Integrated and Experimental Medicine (DIMES), Statistical Service, S. Orsola-Malpighi Hospital Bologna, Bologna, Italy; 8 Department of Anaesthesiology, University Hospital of Modena, Via del Pozzo 71, 41100 Modena, Italy; 9 Anaesthesia and Intensive Care Department, SS. Trinità Hospital, ASL, Novara, Italy; 10 Translational Medicine Department, Eastern Piedmont University, Novara, Italy; 11 Anaesthesia and Intensive Care Residency Program – Translational Medicine Department, Eastern Piedmont University, Novara, Italy; 12 Department of Anaesthesia, Intensive Care and Pain Therapy – Imola Hospital, Imola, Italy; 13 Anaesthesia and Intensive Care Unit, M. Bufalini Hospital, Cesena, Italy; 14 Division of Anesthesiology, Hospital S. Orsola Malpighi, Alma Mater Studiorum University of Bologna, Bologna, Italy; 15 Department of Anesthesia and Intensive Care, G.B. Morgagni-Pierantoni Hospital, Forlì, Italy; 16 Department of Anaesthesia and Intensive Care, Azienda Ospedaliera SS. Antonio e Biagio e Cesare Arrigo, Alessandria, Italy; 17 Department of Anaesthesia and Intensive Care, Infermi Hospital, Rimini, Italy; 18 Anaesthesia and Intensive Care Unit, Santa Maria Annunziata Hospital, Firenze, Italy; 19 Anaesthesia and Intensive Care Unit, Bentivoglio Hospital, Bentivoglio, Italy

**Correction to: J Intensive Care (2020) 8:80**

**https://doi.org/10.1186/s40560-020-00499-4**

Following publication of the original article [1], the authors identified an error in the collaborator author’s name of Elena Mosconi.

The incorrect author name is: Giulia Mosconi

The correct author name is: Elena Mosconi

The original article has been updated.
