# Supplementary material for: Factors influencing liberation from mechanical ventilation in coronavirus disease 2019: multicenter observational study in fifteen Italian ICUs
Source: J Intensive Care. 2020 Oct 15;8:80. doi: 10.1186/s40560-020-00499-4 (PMC7558552; doi:10.1186/s40560-020-00499-4)
Supplement: Supplementary file 1 — Additional file 1:. . [file 40560_2020_499_MOESM1_ESM.zip › Supplement_15_07_CC.docx]

**Factors influencing** **liberation from mechanical ventilation in Coronavirus Disease 2019: multicentre observational study in fifteen Italian ICUs – Supplementary methods and data**

Lorenzo Gamberini MD; Tommaso Tonetti MD; Savino Spadaro MD; Gianluca Zani MD; Carlo Alberto Mazzoli MD; Chiara Capozzi MD; Emanuela Giampalma MD; Maria Letizia Bacchi Reggiani BSc; Elisabetta Bertellini MD; Andrea Castelli MD; Irene Cavalli MD; Davide Colombo MD, PhD; Federico Crimaldi MD; Federica Damiani MD; Alberto Fogagnolo; Maurizio Fusari MD; Emiliano Gamberini MD; Giovanni Gordini MD; Cristiana Laici MD; Maria Concetta Lanza MD; Mirco Leo MD; Andrea Marudi MD; Giuseppe Nardi MD; Irene Ottaviani; Raffaella Papa MD; Antonella Potalivo MD; Emanuele Russo MD; Stefania Taddei MD; Carlo Alberto Volta MD, PhD; V. Marco Ranieri MD, and the ICU-RER COVID-19 Collaboration

***Supplementary methods***

**Collected data**

• Demographics and anamnesis: age, sex, weight, height, previous pathologies, smoker status, therapy with ACE-inhibitors, statins and Angiotensin II Receptor Blockers.

• Conditions at ICU admission: date of symptoms onset, date of hospital admission, date of ICU admission, SOFA and SAPS II score, PaO_2_/FiO_2_ value at the moment of intubation.

• Ventilation during the first 5 days: worst PaO_2_/FiO_2_ value, ventilatory strategy (pressure control ventilation vs volume control ventilation, volumes, lowest pulmonary compliance, worst driving pressure, highest PEEP, highest pCO_2_, number and duration of pronation cycles, response in terms of oxygenation to the first pronation, need for decapneization, use of nitric oxide, tracheostomy date.

• Pharmacologic strategies during the first 5 days: sedative regimen and maximum doses, neuromuscular blocking agents (type of NMBA and duration of continuous infusion).

• COVID specific therapies: antivirals (type, start and end date), chloroquine, tocilizumab (start date and route of administration), intravenous corticosteroids, other specific therapies.

• Other supportive therapies: first line antibacterial regimen, amines (maximum dose), renal replacement therapy, fluidic balance during the first 3 days in ICU

• Complications during ICU stay (see above)

• Weaning from mechanical ventilation: last day of highest PEEP, first attempt of pressure support ventilation (PSV), P/F at the first attempt of PSV, entity of pressure support at the first attempt of PSV, PEEP at the first attempt of PSV, day of extubation, non-invasive ventilation or high flow oxygen therapy after extubation, first day of spontaneous breathing, need for reintubation and date

• Outcome: ICU discharge date, ICU outcome (dead, alive), hospital discharge, 90 days outcome (dead, alive), complications during ICU stay, Quality of life at 90 days (15D instrument)

• Radiology: first available CT, last CT before ICU admission and intubation, last ICU follow-up CT. First available chest X ray, last chest X ray before ICU admission and intubation, last ICU-follow up chest X ray. 30 days follow-up CT (if available).

**Definitions**

*Definition of ventilator-free days (VFDs)*

A time frame of 28 days from intubation was chosen. For intubated patients, in case of interval reintubation within 28 days, VFDs were counted from the last successful extubation. The use of non-invasive ventilation (NIV) after extubation was not considered a ventilation period. Finally, zero VFDs were assigned to 28-day non-survivors, regardless of their intubation status.[1]

For tracheostomized patients, ventilator free days were counted after the last successful disconnection from mechanical ventilation and interval disconnections were not considered as VFDs. [1]

28VFDs are defined as follows:

• VFDs = 0 if subject dies within 28 days of mechanical ventilation;

• VFDs = 28 − x if successfully liberated from ventilation x days after initiation;

• VFDs = 0 if the subject is mechanically ventilated for >28 days.

*Classification and definitions for complications during ICU stay*

Cardiovascular complications

- Myocardial infarction – [2]
  - detection of a rise and/or fall of cTn values with at least 1 value above the 99th percentile and
  - at least 1 of the following:
    - Symptoms of myocardial ischemia;
    - New ischemic ECG changes;
    - Development of pathological Q waves;
    - Imaging evidence of new loss of viable myocardium or new regional wall motion abnormality in a pattern consistent with an ischemic etiology;
    - Identification of a coronary thrombus by angiography or autopsy
- New onset supraventricular or ventricular arrhythmia lasting more than 30 seconds, inducing hemodynamic instability
- Acute pulmonary embolism - Diagnosis obtained Computed tomographic pulmonary angiography [3], irrespective of clinical severity
- Pulmonary oedema
- Haemorrhagic shock - Class III or higher [4]
- Cardiogenic shock - SBP <90 mm Hg with appropriate fluid resuscitation with clinical and laboratory evidence of end‐organ damage Clinical: cold extremities, oliguria, AMS, narrow pulse pressure. Laboratory: metabolic acidosis, elevated serum lactate, elevated serum creatinine [5]
- Septic shock - Sepsis and vasopressor therapy needed to elevate MAP ≥65 mm Hg and lactate >2 mmol/L (18 mg/dL) despite adequate fluid resuscitation [6]
- Acute peripheral ischemia - rapid decrease in limb blood flow due to acute occlusion of peripheral artery or bypass graft needing open surgical revascularization, endovascular, or hybrid approach [7]
- Pneumothorax

Neurologic complications

- Ischemic stroke or cerebral haemorrhage - diagnosed by head computed tomography scan or magnetic resonance imaging [8,9]
- Critical Illness Polyneuropathy/myopathy - confirmed by electrophysiological studies [10]
- New onset seizures - in patients not affected by epilepsy
- Delirium - assessed with CAM-ICU [11]
- Acute gastrointestinal bleeding
- Severe diarrhea - more than 10 loose, watery stools/die
- Gastrointestinal perforation/ischemia [12]
- Bowel obstruction

Renal complications

- Need for renal replacement therapy during ICU stay

Infectious complications

Ventilator associated pneumonia [13]

- - A pneumonia where the patient is on mechanical ventilation for >2 calendar days on the date of event, with day of ventilator placement being Day 1 and the ventilator was in place on the date of event or the day before.
  - Two or more serial chest imaging test results with at least one of the following:
    - New and persistent or Progressive and persistent
    - Infiltrate
    - Consolidation
    - Cavitation
  - At least one of the following:
    - Fever (>38.0°C or >100.4°F) •
    - Leukopenia (≤4000 WBC/mm3) or leukocytosis (>12,000 WBC/mm3)
    - For adults >70 years old, altered mental status with no other recognized cause
  - And at least two of the following:
    - New onset of purulent sputum or change in character of sputum, or increased respiratory secretions, or increased suctioning requirements
    - New onset or worsening cough, or dyspnea, or tachypnea
    - Rales or bronchial breath sounds
    - Worsening gas exchange

VAP was considered of early onset if developed within the first 7 days of mechanical ventilation and late onset if occurred after this period. [14] .

- Bloodstream infection – Primary or secondary - Laboratory-Confirmed Bloodstream Infection Criteria [13]
- Urinary tract infection - Catheter-Associated Urinary Tract Infection and Non-Catheter-Associated Urinary Tract Infection. [13]
- Abdominal Infection -
  - Complicated or uncomplicated abdominal infection - It is broadly defined as peritoneal inflammation in response to microorganisms, resulting in purulence in the peritoneal cavity [15]
  - Clostridium difficile infection [13]

**References**

1. Yehya N, Harhay MO, Curley MAQ, Schoenfeld DA, Reeder RW. Reappraisal of Ventilator-Free Days in Critical Care Research. Am J Respir Crit Care Med. 2019;200:828–36.

2. Thygesen K, Alpert JS, Jaffe AS, Chaitman BR, Bax JJ, Morrow DA, et al. Fourth Universal Definition of Myocardial Infarction (2018). Circulation. 2018.

3. Konstantinides S V., Meyer G, Bueno H, Galié N, Gibbs JSR, Ageno W, et al. 2019 ESC Guidelines for the diagnosis and management of acute pulmonary embolism developed in collaboration with the European respiratory society (ERS). Eur Heart J. 2020;41:543–603.

4. Cannon JW. Hemorrhagic shock. N Engl J Med. 2018;378:370–9.

5. Ponikowski P, Voors AA, Anker SD, Bueno H, Cleland JGF, Coats AJS, et al. 2016 ESC Guidelines for the diagnosis and treatment of acute and chronic heart failure. Eur Heart J. 2016;37:2129-2200m.

6. Singer M, Deutschman CS, Seymour C, Shankar-Hari M, Annane D, Bauer M, et al. The third international consensus definitions for sepsis and septic shock (sepsis-3). JAMA - J Am Med Assoc. 2016;315:801–10.

7. McNally MM, Univers J. Acute Limb Ischemia. Surg Clin North Am. 2018;98:1081–96.

8. Powers WJ, Rabinstein AA, Ackerson T, Adeoye OM, Bambakidis NC, Becker K, et al. 2018 Guidelines for the Early Management of Patients With Acute Ischemic Stroke: A Guideline for Healthcare Professionals From the American Heart Association/American Stroke Association. Stroke. 2018.

9. Runchey S, McGee S. Does this patient have a hemorrhagic stroke? Clinical findings distinguishing hemorrhagic stroke from ischemic stroke. JAMA - J Am Med Assoc. 2010;303:2280–6.

10. Latronico N, Bolton CF. Critical illness polyneuropathy and myopathy: A major cause of muscle weakness and paralysis. Lancet Neurol. Elsevier Ltd; 2011;10:931–41.

11. Figueira Salluh J, Ávila Chalhub R, Quarantini L, Gusmao-Flores D. The confusion assessment method for the intensive care unit (CAM-ICU) and intensive care delirium screening checklist (ICDSC) for the diagnosis of delirium: a systematic review and meta-analysis of clinical studies. Crit Care. 2012;16:2–11.

12. Pouli S, Kozana A, Papakitsou I, Daskalogiannaki M, Raissaki M. Gastrointestinal perforation: clinical and MDCT clues for identification of aetiology. Insights Imaging. Insights into Imaging; 2020;11.

13. CDC, Ncezid, DHQP. National Healthcare Safety Network (NHSN) Patient Safety Component Manual.

14. Giantsou E, Liratzopoulos N, Efraimidou E, Panopoulou M, Alepopoulou E, Kartali-Ktenidou S, et al. Both early-onset and late-onset ventilator-associated pneumonia are caused mainly by potentially multiresistant bacteria. Intensive Care Med. 2005;31:1488–94.

15. Lopez N, Kobayashi L, Coimbra R. A Comprehensive review of abdominal infections. World J Emerg Surg. BioMed Central Ltd; 2011;6:7.

**Supplement Tables**

Table S1. Adjunctive therapies.

| Adjunctive treatments | n = 391 |
| --- | --- |
| Neuromuscular blocking agents continuous infusion - no (%) | 365 (93.4%) |
| Prone positioning - no (%) | 258 (66.0%) |
| Prone positioning cycles - no (IQR) | 3 (2-4) |
| Prone positioning cycle duration - hours (IQR) | 16 (16 - 17) |
| PaO2/FiO2 ratio before first prone positioning (n = 234) *^a^* - (IQR) | 113 (83 - 128) |
| PaO2/FiO2 ratio after first prone positioning (n = 226) *^a^* - (IQR) | 123 (124 - 228) |
| Inhaled nitric oxide (iNO) - no (%) | 20 (5.1%) |
|  |  |
| COVID-19 supportive therapies | n = 391 |
| Tocilizumab - no (%) | 187 (47.8%) |
| - Intravenous - no (%) | 107 (57.2%) |
| - Subcutaneous - no (%) | 61 (32.6%) |
| - Subcutaneous and intravenous - no (%) | 19 (10.2%) |
| Canakinumab - no (%) | 26 (6.6%) |
| Corticosteroids - no (%) | 264 (67.5%) |
| Chloroquine/hydroxychloroquine - no (%) | 376 (96.2%) |
| Antiviral drugs |  |
| - Lopinavir-Ritonavir - no (%) | 117 (29.9%) |
| - Darunavir - Ritonavir - no (%) | 32 (8.2%) |
| - Darunavir - Cobicistat - no (%) | 123 (31.5%) |
| - Remdesivir - no (%) | 9 (2.3%) |
| - Oseltamivir - no (%) | 5 (1.3%) |
|  |  |
| Other drugs | n = 391 |
| Antiplatelet therapy - no (%) | 91 (23.3%) |
| Anticoagulation - no (%) | 385 (98.5%) |
| Anticoagulation - LMWH - no (%) | 364 (93.1%) |
| Anticoagulation - calcium heparin - no (%) | 20 (5.1%) |
| Vasoactive drugs - no (%) | 278 (71.1%) |
| - Norepinephrine - no (%) | 271 (69.3.%) |
| - Epinephrine - no (%) | 24 (6.1%) |
| - Dopamine - no (%) | 20 (5.1%) |
| - Dobutamine - no (%) | 36 (9.2%) |
| - Other - no (%) | 5 (1.3%) |

Notes:

*^a^* incomplete data due to transfer from other ICUs without complete medical records.

Table S2. Main complications during ICU stay.

| Complications | n=391 |
| --- | --- |
|  |  |
| Infections | n=238 (60.9%) |
| Ventilator associated pneumonia |  |
| - Early onset VAP - no (%) | 76 (19.4%). |
| - Late onset VAP - no (%) | 175 (44.8%) |
|  |  |
| Non-pulmonary sepsis - no (%) | 112 (28.6%) |
| - Bloodstream infection - no (%) | 107 (27.4%) |
| - Urinary tract infection - no (%) | 8 (2.0%) |
| - Abdominal infection - no (%) | 6 (1.5%) |
|  |  |
| Other complications | 201 (51.4%) |
| Cardiovascular - no (%) | 66 (16.9%) |
| - Myocardial infarction - no (%) | 3 (0.8%) |
| - New onset supraventricular or ventricular arrhythmia - no (%) | 26 (6.6%) |
| - Pulmonary embolism - no (%) | 15 (3.8%) |
| - Pulmonary oedema - no (%) | 0 (0%) |
| - Haemorrhagic shock - no (%) | 5 (1.3%) |
| - Cardiogenic shock - no (%) | 8 (2.0%) |
| - Septic shock - no (%) | 31 (7.9%) |
| - Acute peripheral ischemia - no (%) | 3 (0.8%) |
| - Pneumothorax - no (%) | 19 (4.9%) |
|  |  |
| Neurologic - no (%) | 25 (6.4%) |
| - Ischemic stroke or cerebral haemorrhage - no (%) | 4 (1%) |
| - Critical Illness Polyneuropathy/myopathy - no (%) | 15 (3.8%) |
| - New onset seizures - no (%) | 1 (0.3%) |
| - Delirium - no (%) | 10 (2.6%) |
|  |  |
| Gastroenteric - no (%) | 20 (5.1%) |
| - Gastrointestinal bleeding - no (%) | 5 (1.3%) |
| - Severe diarrhea (> 10 loose, watery stools/die) - no (%) | 12 (3.0%) |
| - Gastrointestinal perforation/ischemia - no (%) | 4 (1%) |
| - Bowel Obstruction - no (%) | 1 (0.3%) |
|  |  |
| Need for renal replacement therapy - no (%) | 76 (19.4%) |

**Supplement Figures**

Figure S1 – Patients’ flow through the screening and enrolment process and number of patients enrolled per centre

Note - Center codes: 1 - Sant'Antonio e Biagio e Cesare Arrigo Hospital, Alessandria, IT; 2 – Sant’Orsola-Malpighi hospital pavilions 23-25, Bologna, IT;3 - Sant’Orsola-Malpighi hospital pavilion 5, Bologna, IT; 4 – Maggiore Hospital Carlo Alberto Pizzardi, Bologna, IT; 5 – Bellaria Hospital, Bologna, IT; 6 – Santa Maria della Scaletta Hospital, Imola, IT; 7 – Bentivoglio Hospital, Bentivoglio, IT; 8 - Bufalini Hospital, Cesena, IT;9 - Morgagni-Pierantoni Hospital, Forlì, IT; 10 – Arcispedale Sant’Anna, Ferrara, IT; 11 – Santa Maria Annunziata hospital, Firenze, IT; 12 – Ospedale Civile di Baggiovara, Modena, IT; 13 - SS. Trinità Hospital, Borgomanero, IT; 14 - S. Maria delle Croci Hospital, Ravenna, IT; 15 – Infermi Hospital, Rimini, IT.

Figure S2 – ROC curve graph for the multivariate model for logistic regression described in table 4
