# Supplementary figures and images for: Factors influencing liberation from mechanical ventilation in coronavirus disease 2019: multicenter observational study in fifteen Italian ICUs
Source: J Intensive Care. 2020 Oct 15;8:80. doi: 10.1186/s40560-020-00499-4 (PMC7558552; doi:10.1186/s40560-020-00499-4)

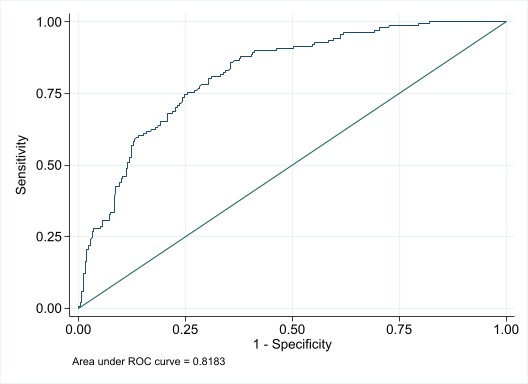

Supplement: Supplementary file 1 — Additional file 1:. . [file 40560_2020_499_MOESM1_ESM.zip › Figure S2.tiff]
